# Poly[dimethyl­ammonium [aquadi-μ_2_-oxalato-samarate(III)] trihydrate]

**DOI:** 10.1107/S1600536811020058

**Published:** 2011-06-04

**Authors:** Yao-Kang Lv, Li-Hua Gan, Ming-Xian Liu, Wei Xiong

**Affiliations:** a Department of Chemistry, Tongji University, Shanghai 200092, People’s Republic of China

## Abstract

In the title complex, {(C_2_H_8_N)[Sm(C_2_O_4_)_2_(H_2_O)]·3H_2_O}_*n*_, the Sm^III^ atom is chelated by four oxalate ligands and one water mol­ecule forming a distorted tricapped trigonal–prismatic geometry. Each oxalate ligand chelates to two Sm^III^ atoms, generating a three-dimensional anionic network with cavities in which the ammonium cations and lattice water mol­ecules reside. Various O—H⋯O, N—H⋯O and C—H⋯O hydrogen-bonding inter­actions further stablize the crystal structure.

## Related literature

For general background to the rational design and synthesis of metal-organic polymers, see: Kim *et al.* (1998 ▶); Lv *et al.* (2011 ▶). For related structures, see: Lv *et al.* (2010 ▶); Trombe & Mohanu (2004 ▶). The structure of the isotypic Eu^III^ compound was reported by Yang *et al.* (2005 ▶), and the Dy^III^ compound was reported by Ye & Lin (2010 ▶).
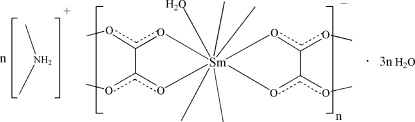


## Experimental

### 

#### Crystal data

(C_2_H_8_N)[Sm(C_2_O_4_)_2_(H_2_O)]·3H_2_O
*M* *_r_* = 444.55
Monoclinic, 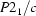

*a* = 9.6711 (3) Å
*b* = 11.7849 (3) Å
*c* = 14.3863 (4) Å
β = 122.276 (2)°
*V* = 1386.30 (7) Å^3^
*Z* = 4
Mo *K*α radiation
μ = 4.30 mm^−1^
*T* = 296 K
0.17 × 0.14 × 0.08 mm

#### Data collection

Bruker APEXII area-detector diffractometer
Absorption correction: multi-scan (*SADABS*; Sheldrick, 1996 ▶) *T*_min_ = 0.36, *T*_max_ = 0.43
12866 measured reflections
3225 independent reflections
2739 reflections with *I* > 2σ(*I*)
*R*_int_ = 0.039

#### Refinement

*R*[*F* ^2^ > 2σ(*F* ^2^)] = 0.026
*wR*(*F* ^2^) = 0.061
*S* = 1.03
3225 reflections
207 parameters
12 restraints
H atoms treated by a mixture of independent and constrained refinement
Δρ_max_ = 0.69 e Å^−3^
Δρ_min_ = −0.98 e Å^−3^

### 

Data collection: *APEX2* (Bruker, 2006 ▶); cell refinement: *SAINT* (Bruker, 2006 ▶); data reduction: *SAINT*; program(s) used to solve structure: *SHELXS97* (Sheldrick, 2008 ▶); program(s) used to refine structure: *SHELXL97* (Sheldrick, 2008 ▶); molecular graphics: *DIAMOND* (Brandenburg & Putz, 2004 ▶); software used to prepare material for publication: *SHELXTL* (Sheldrick, 2008 ▶).

## Supplementary Material

Crystal structure: contains datablock(s) global, I. DOI: 10.1107/S1600536811020058/om2429sup1.cif

Structure factors: contains datablock(s) I. DOI: 10.1107/S1600536811020058/om2429Isup2.hkl

Additional supplementary materials:  crystallographic information; 3D view; checkCIF report

## Figures and Tables

**Table 1:** Hydrogen-bond geometry (Å, °)

*D*—H⋯*A*	*D*—H	H⋯*A*	*D*⋯*A*	*D*—H⋯*A*
O1*W*—H1*WA*⋯O2*W*^i^	0.82 (2)	1.95 (2)	2.764 (5)	174 (5)
O1*W*—H1*WB*⋯O2*W*^ii^	0.83 (2)	2.03 (2)	2.852 (5)	172 (4)
O2*W*—H2*WA*⋯O6^iii^	0.81 (2)	2.26 (2)	3.065 (5)	175 (6)
O2*W*—H2*WA*⋯O7^iv^	0.81 (2)	2.47 (5)	3.023 (5)	126 (5)
O2*W*—H2*WB*⋯O4*W*	0.80 (2)	2.51 (2)	3.306 (7)	179 (6)
O2*W*—H2*WB*⋯O3*W*^v^	0.80 (2)	2.64 (5)	3.039 (8)	113 (5)
O3*W*—H3*WA*⋯O2	0.84 (2)	2.08 (3)	2.845 (5)	151 (6)
O3*W*—H3*WB*⋯O4*W*^iv^	0.82 (2)	1.98 (2)	2.796 (6)	172 (8)
O4*W*—H4*WA*⋯O1^vi^	0.83 (2)	2.15 (2)	2.967 (5)	169 (6)
O4*W*—H4*WB*⋯O3^vii^	0.82 (2)	2.11 (2)	2.898 (5)	160 (6)
N1—H1*A*⋯O3*W*	0.90	1.85	2.744 (6)	171
N1—H1*B*⋯O8^vi^	0.90	1.99	2.864 (5)	163
N1—H1*B*⋯O1*W*^vi^	0.90	2.50	3.071 (5)	122
C5—H5*C*⋯O4^iii^	0.96	2.53	3.283 (7)	135

Symmetry codes: (i) 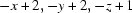; (ii) 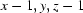; (iii) 
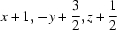
; (iv) 
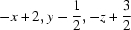
; (v) 
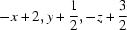
; (vi) 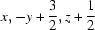; (vii) 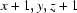.

**Fig. 1.**
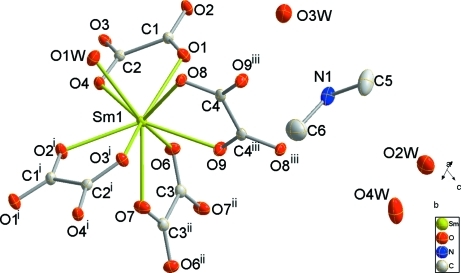
Structure of the title compound showing the atom labeling, thermal displacement parameters are shown at the 30% probability level. All the hydrogen atoms are omitted for clarity. [Symmetry code: (i) -x + 1, y + 1/2, -z + 1/2 (ii) -x + 1, -y + 2, -z + 1; (iii) -x + 2, -y + 2, -z + 1.]

**Fig. 2.**
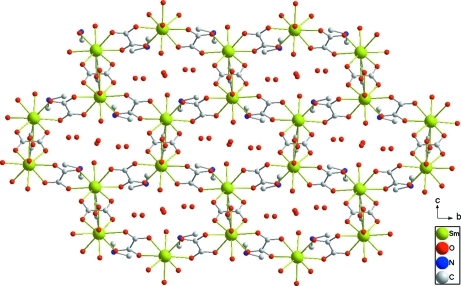
View of the three-dimensional framework. All the hydrogen atoms are omitted for clarity.

## supplementary crystallographic information

### Comment

Carbohydrates and their derivatives are the most widely distributed naturally occurring compounds and are indispensable to living organisms as an energy source and building blocks. Many carbohydrate-derived ligands were prepared and occasionally lead to novel and versatile metal-organic polymers with interesting structures (Kim *et al.*, 1998; Lv *et al.*, 2011). Oxalate, which usually represent one of the products of the degradation of carbohydrates, is one of the simplest multidentate organic ligands potentially able to bridge metal ions in a bidentate chelating manner (Lv *et al.*, 2010; Trombe & Mohanu, 2004). We report herein the synthesis and structure of a new samarium(III) complex, (C_2_H_8_N)[Sm(C_2_O_4_)_2_(H_2_O)].3H_2_O. The oxalate ligands in this complex arise from the decomposition of potassium hydrogen saccharate.

Single-crystal X-ray diffraction analysis revealed that the title complex crystallizes in the monoclinic system with space group *P*2_1_/*c*, and is isostructural with its Eu^III^ analogue (Yang *et al.*, 2005) and Dy^III^ analogues (Ye & Lin, 2010). As shown in Fig. 1, the Sm^III^ atom is chelated by four oxalate ligands and one water molecule forming a distorted tricapped trigonal-prismatic geometry. Each oxalate ligand bridges two Sm^III^ atoms, generating a three-dimensional anionic network with cavities where the ammonium cations and lattice water molecules reside (Fig. 2). Furthermore, there are various hydrogen-bonding interactions (N—H···O, O—H···O and C—H···O) stablizing the crystal framework.

### Experimental

A mixture of potassium hydrogen saccharate (0.248 g, 1.0 mmol), Sm(NO_3_)_3_.6H_2_O (0.222 g, 0.5 mmol) and N, *N*-Dimethylformamide (20 ml) was stirred and heated at 373 K for 60 minute, the resulted colorless solution was kept at 20°C. Colorless block crystals suitable for X-ray crystallographic study were crystralized *via* slow evaporation in 2 weeks.

### Refinement

The structure was solved by direct methods and expanded with difference Fourier techniques. All non-hydrogen atoms were refined anisotropically by the full matrix least-squares on the F2. The hydrogen atoms attached to carbon and nitrogen atoms were located by geometrical calculation and included in the refinement using a riding model [C—H 0.96 Å and N—H 0.90 Å], while those attached to oxygen atom were located from the difference Fourier maps and their positions were refined with O—H distances fixed at 0.82 (5) Å.

### Crystal data

**Table:**

(C_2_H_8_N)[Sm(C_2_O_4_)_2_(H_2_O)]·3H_2_O	*F*(000) = 868
*M**_r_* = 444.55	*D*_x_ = 2.130 Mg m^−^^3^
Monoclinic, *P*2_1_/*c*	Mo *K*α radiation, λ = 0.71073 Å
Hall symbol: -P 2ybc	Cell parameters from 3706 reflections
*a* = 9.6711 (3) Å	θ = 2.5–27.7°
*b* = 11.7849 (3) Å	µ = 4.30 mm^−^^1^
*c* = 14.3863 (4) Å	*T* = 296 K
β = 122.276 (2)°	Block, colourless
*V* = 1386.30 (7) Å^3^	0.17 × 0.14 × 0.08 mm
*Z* = 4	

### Data collection

**Table:**

Bruker APEXII area-detector diffractometer	3225 independent reflections
Radiation source: fine-focus sealed tube	2739 reflections with *I* > 2σ(*I*)
graphite	*R*_int_ = 0.039
ω scans	θ_max_ = 27.7°, θ_min_ = 2.5°
Absorption correction: multi-scan (*SADABS*; Sheldrick, 1996)	*h* = −12→12
*T*_min_ = 0.36, *T*_max_ = 0.43	*k* = −15→14
12866 measured reflections	*l* = −16→18

### Refinement

**Table:**

Refinement on *F*^2^	Primary atom site location: structure-invariant direct methods
Least-squares matrix: full	Secondary atom site location: difference Fourier map
*R*[*F*^2^ > 2σ(*F*^2^)] = 0.026	Hydrogen site location: inferred from neighbouring sites
*wR*(*F*^2^) = 0.061	H atoms treated by a mixture of independent and constrained refinement
*S* = 1.03	*w* = 1/[σ^2^(*F*_o_^2^) + (0.0215*P*)^2^ + 2.7236*P*] where *P* = (*F*_o_^2^ + 2*F*_c_^2^)/3
3225 reflections	(Δ/σ)_max_ = 0.001
207 parameters	Δρ_max_ = 0.69 e Å^−^^3^
12 restraints	Δρ_min_ = −0.98 e Å^−^^3^

### Special details

**Table:**

Geometry. All e.s.d.'s (except the e.s.d. in the dihedral angle between two l.s. planes) are estimated using the full covariance matrix. The cell e.s.d.'s are taken into account individually in the estimation of e.s.d.'s in distances, angles and torsion angles; correlations between e.s.d.'s in cell parameters are only used when they are defined by crystal symmetry. An approximate (isotropic) treatment of cell e.s.d.'s is used for estimating e.s.d.'s involving l.s. planes.
Refinement. Refinement of *F*^2^ against ALL reflections. The weighted *R*-factor *wR* and goodness of fit *S* are based on *F*^2^, conventional *R*-factors *R* are based on *F*, with *F* set to zero for negative *F*^2^. The threshold expression of *F*^2^ > σ(*F*^2^) is used only for calculating *R*-factors(gt) *etc*. and is not relevant to the choice of reflections for refinement. *R*-factors based on *F*^2^ are statistically about twice as large as those based on *F*, and *R*- factors based on ALL data will be even larger.

### Fractional atomic coordinates and isotropic or equivalent isotropic displacement parameters (Å^2^)

**Table:**

	*x*	*y*	*z*	*U*_iso_*/*U*_eq_	
Sm1	0.61680 (2)	0.983582 (14)	0.330364 (15)	0.01793 (7)	
O1	0.7036 (3)	0.7832 (2)	0.3435 (2)	0.0284 (6)	
O2	0.6156 (3)	0.6045 (2)	0.3014 (2)	0.0293 (7)	
O3	0.3063 (3)	0.6799 (2)	0.1732 (2)	0.0242 (6)	
O4	0.3978 (3)	0.8576 (2)	0.2047 (2)	0.0241 (6)	
O6	0.5172 (3)	0.8817 (2)	0.4343 (2)	0.0286 (6)	
O7	0.5330 (4)	1.1081 (2)	0.4324 (3)	0.0334 (7)	
O8	0.8912 (3)	0.9847 (2)	0.3572 (2)	0.0253 (6)	
O9	0.8415 (3)	0.9876 (2)	0.5228 (2)	0.0289 (6)	
O1W	0.6084 (4)	0.9755 (3)	0.1530 (2)	0.0330 (7)	
H1WA	0.608 (6)	1.033 (2)	0.121 (3)	0.040*	
H1WB	0.544 (5)	0.932 (3)	0.103 (3)	0.040*	
O2W	1.3864 (6)	0.8423 (3)	0.9661 (4)	0.0614 (11)	
H2WA	1.426 (7)	0.783 (3)	0.962 (5)	0.074*	
H2WB	1.306 (5)	0.827 (5)	0.966 (6)	0.074*	
O3W	0.8362 (7)	0.5053 (4)	0.5101 (4)	0.0799 (15)	
H3WA	0.793 (9)	0.521 (5)	0.443 (2)	0.096*	
H3WB	0.876 (8)	0.442 (3)	0.517 (5)	0.096*	
O4W	1.0543 (5)	0.7816 (4)	0.9682 (4)	0.0767 (14)	
H4WA	0.958 (3)	0.763 (6)	0.942 (5)	0.092*	
H4WB	1.106 (6)	0.742 (5)	1.024 (4)	0.092*	
N1	0.9284 (5)	0.6293 (4)	0.6960 (3)	0.0430 (10)	
H1A	0.8867	0.5890	0.6334	0.052*	
H1B	0.8947	0.5962	0.7369	0.052*	
C1	0.5958 (4)	0.7095 (3)	0.2949 (3)	0.0212 (8)	
C2	0.4172 (4)	0.7536 (3)	0.2173 (3)	0.0200 (7)	
C3	0.4950 (4)	0.9348 (3)	0.5004 (3)	0.0238 (8)	
C4	1.0168 (4)	0.9999 (3)	0.4541 (3)	0.0202 (8)	
C5	1.1056 (7)	0.6231 (7)	0.7555 (5)	0.076 (2)	
H5A	1.1392	0.5450	0.7687	0.091*	
H5B	1.1514	0.6621	0.8243	0.091*	
H5C	1.1433	0.6579	0.7126	0.091*	
C6	0.8628 (9)	0.7443 (6)	0.6685 (6)	0.083 (2)	
H6A	0.7455	0.7414	0.6256	0.100*	
H6B	0.9012	0.7813	0.6269	0.100*	
H6C	0.8987	0.7861	0.7349	0.100*	

### Atomic displacement parameters (Å^2^)

**Table:**

	*U*^11^	*U*^22^	*U*^33^	*U*^12^	*U*^13^	*U*^23^
Sm1	0.01682 (10)	0.01446 (10)	0.01891 (10)	−0.00015 (7)	0.00713 (8)	−0.00032 (7)
O1	0.0186 (14)	0.0194 (14)	0.0365 (17)	−0.0004 (10)	0.0076 (12)	−0.0044 (11)
O2	0.0225 (14)	0.0156 (13)	0.0327 (16)	0.0014 (10)	0.0032 (12)	−0.0002 (11)
O3	0.0199 (13)	0.0176 (13)	0.0313 (15)	0.0008 (10)	0.0111 (12)	−0.0021 (11)
O4	0.0222 (14)	0.0171 (13)	0.0234 (14)	0.0010 (10)	0.0057 (12)	0.0003 (10)
O6	0.0358 (16)	0.0225 (14)	0.0311 (16)	−0.0052 (11)	0.0203 (13)	−0.0054 (11)
O7	0.0479 (18)	0.0213 (14)	0.0428 (18)	0.0052 (12)	0.0322 (16)	0.0063 (12)
O8	0.0199 (13)	0.0337 (15)	0.0169 (13)	−0.0009 (11)	0.0061 (11)	−0.0030 (11)
O9	0.0195 (14)	0.0437 (17)	0.0235 (14)	−0.0032 (12)	0.0114 (12)	−0.0030 (12)
O1W	0.0363 (17)	0.0349 (17)	0.0209 (15)	−0.0056 (13)	0.0108 (13)	−0.0013 (12)
O2W	0.073 (3)	0.035 (2)	0.063 (3)	0.0036 (18)	0.028 (2)	0.0070 (19)
O3W	0.084 (3)	0.078 (3)	0.047 (3)	0.027 (3)	0.014 (3)	−0.003 (2)
O4W	0.056 (3)	0.053 (3)	0.069 (3)	−0.008 (2)	−0.002 (2)	0.014 (2)
N1	0.035 (2)	0.057 (3)	0.036 (2)	−0.0080 (18)	0.0180 (18)	0.0077 (18)
C1	0.0196 (19)	0.0201 (19)	0.0190 (18)	0.0007 (13)	0.0069 (16)	−0.0017 (14)
C2	0.0209 (19)	0.0206 (19)	0.0171 (17)	0.0011 (14)	0.0092 (15)	0.0001 (14)
C3	0.0196 (18)	0.025 (2)	0.0225 (19)	0.0000 (14)	0.0081 (15)	0.0006 (15)
C4	0.0217 (19)	0.0137 (17)	0.0190 (18)	−0.0016 (13)	0.0068 (15)	0.0015 (13)
C5	0.041 (3)	0.126 (6)	0.051 (4)	−0.010 (4)	0.018 (3)	0.020 (4)
C6	0.104 (6)	0.067 (4)	0.086 (5)	0.012 (4)	0.056 (5)	0.017 (4)

### Geometric parameters (Å, °)

**Table:**

Sm1—O4	2.422 (3)	O2W—H2WA	0.811 (19)
Sm1—O3^i^	2.440 (2)	O2W—H2WB	0.798 (19)
Sm1—O9	2.442 (3)	O3W—H3WA	0.839 (19)
Sm1—O8	2.469 (3)	O3W—H3WB	0.819 (19)
Sm1—O2^i^	2.474 (3)	O4W—H4WA	0.83 (2)
Sm1—O6	2.479 (3)	O4W—H4WB	0.823 (19)
Sm1—O1	2.479 (3)	N1—C5	1.452 (7)
Sm1—O7	2.497 (3)	N1—C6	1.459 (8)
Sm1—O1W	2.512 (3)	N1—H1A	0.9001
O1—C1	1.246 (4)	N1—H1B	0.9001
O2—C1	1.248 (4)	C1—C2	1.562 (5)
O2—Sm1^ii^	2.474 (3)	C3—O7^iii^	1.242 (5)
O3—C2	1.257 (4)	C3—C3^iii^	1.541 (8)
O3—Sm1^ii^	2.440 (2)	C4—O9^iv^	1.234 (5)
O4—C2	1.238 (4)	C4—C4^iv^	1.519 (8)
O6—C3	1.248 (5)	C5—H5A	0.9600
O7—C3^iii^	1.242 (5)	C5—H5B	0.9600
O8—C4	1.282 (4)	C5—H5C	0.9600
O9—C4^iv^	1.234 (5)	C6—H6A	0.9600
O1W—H1WA	0.817 (18)	C6—H6B	0.9600
O1W—H1WB	0.828 (18)	C6—H6C	0.9600
			
O4—Sm1—O3^i^	136.09 (9)	C3—O6—Sm1	119.8 (2)
O4—Sm1—O9	138.78 (9)	C3^iii^—O7—Sm1	119.5 (2)
O3^i^—Sm1—O9	84.76 (9)	C4—O8—Sm1	119.1 (2)
O4—Sm1—O8	123.92 (9)	C4^iv^—O9—Sm1	119.7 (2)
O3^i^—Sm1—O8	71.56 (9)	Sm1—O1W—H1WA	122 (3)
O9—Sm1—O8	65.88 (9)	Sm1—O1W—H1WB	120 (3)
O4—Sm1—O2^i^	72.94 (8)	H1WA—O1W—H1WB	104 (3)
O3^i^—Sm1—O2^i^	66.43 (8)	H2WA—O2W—H2WB	108 (3)
O9—Sm1—O2^i^	138.73 (9)	H3WA—O3W—H3WB	105 (3)
O8—Sm1—O2^i^	125.24 (9)	H4WA—O4W—H4WB	105 (3)
O4—Sm1—O6	71.76 (9)	C5—N1—C6	114.3 (5)
O3^i^—Sm1—O6	133.88 (9)	C5—N1—H1A	108.6
O9—Sm1—O6	73.91 (9)	C6—N1—H1A	108.8
O8—Sm1—O6	129.79 (9)	C5—N1—H1B	108.5
O2^i^—Sm1—O6	104.74 (10)	C6—N1—H1B	108.8
O4—Sm1—O1	66.34 (8)	H1A—N1—H1B	107.6
O3^i^—Sm1—O1	144.12 (9)	O1—C1—O2	126.9 (3)
O9—Sm1—O1	82.65 (9)	O1—C1—C2	116.3 (3)
O8—Sm1—O1	72.62 (9)	O2—C1—C2	116.8 (3)
O2^i^—Sm1—O1	137.62 (9)	O4—C2—O3	126.0 (3)
O6—Sm1—O1	73.62 (9)	O4—C2—C1	117.2 (3)
O4—Sm1—O7	111.72 (10)	O3—C2—C1	116.8 (3)
O3^i^—Sm1—O7	69.72 (9)	O7^iii^—C3—O6	125.9 (4)
O9—Sm1—O7	72.18 (10)	O7^iii^—C3—C3^iii^	116.9 (4)
O8—Sm1—O7	124.34 (9)	O6—C3—C3^iii^	117.2 (4)
O2^i^—Sm1—O7	70.42 (10)	O9^iv^—C4—O8	125.5 (4)
O6—Sm1—O7	64.99 (9)	O9^iv^—C4—C4^iv^	119.2 (4)
O1—Sm1—O7	135.89 (10)	O8—C4—C4^iv^	115.3 (4)
O4—Sm1—O1W	71.17 (10)	N1—C5—H5A	109.5
O3^i^—Sm1—O1W	81.92 (10)	N1—C5—H5B	109.5
O9—Sm1—O1W	132.76 (10)	H5A—C5—H5B	109.5
O8—Sm1—O1W	66.90 (9)	N1—C5—H5C	109.5
O2^i^—Sm1—O1W	73.75 (10)	H5A—C5—H5C	109.5
O6—Sm1—O1W	141.50 (9)	H5B—C5—H5C	109.5
O1—Sm1—O1W	82.35 (10)	N1—C6—H6A	109.5
O7—Sm1—O1W	140.82 (10)	N1—C6—H6B	109.5
C1—O1—Sm1	118.3 (2)	H6A—C6—H6B	109.5
C1—O2—Sm1^ii^	118.2 (2)	N1—C6—H6C	109.5
C2—O3—Sm1^ii^	118.6 (2)	H6A—C6—H6C	109.5
C2—O4—Sm1	119.6 (2)	H6B—C6—H6C	109.5
			
O4—Sm1—O1—C1	−10.9 (3)	O4—Sm1—O8—C4	−141.7 (2)
O3^i^—Sm1—O1—C1	−148.3 (3)	O3^i^—Sm1—O8—C4	84.6 (2)
O9—Sm1—O1—C1	141.3 (3)	O9—Sm1—O8—C4	−7.9 (2)
O8—Sm1—O1—C1	−151.8 (3)	O2^i^—Sm1—O8—C4	125.8 (2)
O2^i^—Sm1—O1—C1	−28.1 (4)	O6—Sm1—O8—C4	−47.7 (3)
O6—Sm1—O1—C1	66.0 (3)	O1—Sm1—O8—C4	−97.6 (3)
O7—Sm1—O1—C1	86.5 (3)	O7—Sm1—O8—C4	36.7 (3)
O1W—Sm1—O1—C1	−83.6 (3)	O1W—Sm1—O8—C4	173.5 (3)
O3^i^—Sm1—O4—C2	158.7 (3)	O4—Sm1—O9—C4^iv^	122.0 (3)
O9—Sm1—O4—C2	−31.0 (3)	O3^i^—Sm1—O9—C4^iv^	−64.8 (3)
O8—Sm1—O4—C2	60.2 (3)	O8—Sm1—O9—C4^iv^	7.4 (3)
O2^i^—Sm1—O4—C2	−178.4 (3)	O2^i^—Sm1—O9—C4^iv^	−109.3 (3)
O6—Sm1—O4—C2	−66.0 (3)	O6—Sm1—O9—C4^iv^	156.6 (3)
O1—Sm1—O4—C2	13.6 (3)	O1—Sm1—O9—C4^iv^	81.6 (3)
O7—Sm1—O4—C2	−118.4 (3)	O7—Sm1—O9—C4^iv^	−135.1 (3)
O1W—Sm1—O4—C2	103.4 (3)	O1W—Sm1—O9—C4^iv^	9.2 (3)
O4—Sm1—O6—C3	−136.1 (3)	Sm1—O1—C1—O2	−172.2 (3)
O3^i^—Sm1—O6—C3	1.2 (3)	Sm1—O1—C1—C2	8.1 (4)
O9—Sm1—O6—C3	67.1 (3)	Sm1^ii^—O2—C1—O1	−169.5 (3)
O8—Sm1—O6—C3	104.5 (3)	Sm1^ii^—O2—C1—C2	10.2 (5)
O2^i^—Sm1—O6—C3	−70.1 (3)	Sm1—O4—C2—O3	165.1 (3)
O1—Sm1—O6—C3	154.0 (3)	Sm1—O4—C2—C1	−15.0 (4)
O7—Sm1—O6—C3	−10.4 (3)	Sm1^ii^—O3—C2—O4	162.8 (3)
O1W—Sm1—O6—C3	−152.4 (3)	Sm1^ii^—O3—C2—C1	−17.2 (4)
O4—Sm1—O7—C3^iii^	66.9 (3)	O1—C1—C2—O4	4.4 (5)
O3^i^—Sm1—O7—C3^iii^	−160.3 (3)	O2—C1—C2—O4	−175.4 (4)
O9—Sm1—O7—C3^iii^	−69.4 (3)	O1—C1—C2—O3	−175.7 (3)
O8—Sm1—O7—C3^iii^	−111.7 (3)	O2—C1—C2—O3	4.5 (5)
O2^i^—Sm1—O7—C3^iii^	128.4 (3)	Sm1—O6—C3—O7^iii^	−169.7 (3)
O6—Sm1—O7—C3^iii^	10.8 (3)	Sm1—O6—C3—C3^iii^	9.6 (5)
O1—Sm1—O7—C3^iii^	−11.0 (4)	Sm1—O8—C4—O9^iv^	−173.2 (3)
O1W—Sm1—O7—C3^iii^	153.4 (3)	Sm1—O8—C4—C4^iv^	7.9 (5)

Symmetry codes: (i) −*x*+1, *y*+1/2, −*z*+1/2; (ii) −*x*+1, *y*−1/2, −*z*+1/2; (iii) −*x*+1, −*y*+2, −*z*+1; (iv) −*x*+2, −*y*+2, −*z*+1.

### Hydrogen-bond geometry (Å, °)

**Table:**

*D*—H···*A*	*D*—H	H···*A*	*D*···*A*	*D*—H···*A*
O1W—H1WA···O2W^iv^	0.82 (2)	1.95 (2)	2.764 (5)	174 (5)
O1W—H1WB···O2W^v^	0.83 (2)	2.03 (2)	2.852 (5)	172 (4)
O2W—H2WA···O6^vi^	0.81 (2)	2.26 (2)	3.065 (5)	175 (6)
O2W—H2WA···O7^vii^	0.81 (2)	2.47 (5)	3.023 (5)	126 (5)
O2W—H2WB···O4W	0.80 (2)	2.51 (2)	3.306 (7)	179 (6)
O2W—H2WB···O3W^viii^	0.80 (2)	2.64 (5)	3.039 (8)	113 (5)
O3W—H3WA···O2	0.84 (2)	2.08 (3)	2.845 (5)	151 (6)
O3W—H3WB···O4W^vii^	0.82 (2)	1.98 (2)	2.796 (6)	172 (8)
O4W—H4WA···O1^ix^	0.83 (2)	2.15 (2)	2.967 (5)	169 (6)
O4W—H4WB···O3^x^	0.82 (2)	2.11 (2)	2.898 (5)	160 (6)
N1—H1A···O3W	0.90	1.85	2.744 (6)	171
N1—H1B···O8^ix^	0.90	1.99	2.864 (5)	163
N1—H1B···O1W^ix^	0.90	2.50	3.071 (5)	122
C5—H5C···O4^vi^	0.96	2.53	3.283 (7)	135

Symmetry codes: (iv) −*x*+2, −*y*+2, −*z*+1; (v) *x*−1, *y*, *z*−1; (vi) *x*+1, −*y*+3/2, *z*+1/2; (vii) −*x*+2, *y*−1/2, −*z*+3/2; (viii) −*x*+2, *y*+1/2, −*z*+3/2; (ix) *x*, −*y*+3/2, *z*+1/2; (x) *x*+1, *y*, *z*+1.
